# Assessment of the Functional Capacity and Preparedness of the Haitian Healthcare System to Fight against the COVID-19 Pandemic: A Narrative Review

**DOI:** 10.3390/healthcare10081428

**Published:** 2022-07-29

**Authors:** Josemyrne Ashley Faure, Chia-Wen Wang, Chi-Hsin Sally Chen, Chang-Chuan Chan

**Affiliations:** 1 Global Health Program, College of Public Health, National Taiwan University, No. 17, Xu-Zhou Rd., Taipei 10055, Taiwan; fashleyjosemyrne@gmail.com; 2 Innovation and Policy Centre for Population Health and Sustainable Environment (Population Health Research Centre, PHRC), College of Public Health, National Taiwan University, No. 17, Xu-Zhou Rd., Taipei 10055, Taiwan; am10312002@gmail.com; 3 Institute of Environmental and Occupational Health Sciences, College of Public Health, National Taiwan University, No. 17, Xu-Zhou Rd., Taipei 10055, Taiwan; chschen@ntu.edu.tw

**Keywords:** COVID-19, Haitian healthcare, healthcare system preparedness, pandemics

## Abstract

Low-income countries, such as Haiti, are facing challenges in fighting the COVID-19 pandemic due to resource shortages and fragile healthcare systems. This study assessed the functional capacity and preparedness of the Haitian healthcare system regarding the COVID-19 pandemic. It employed a narrative review approach to analyze secondary data and used the Donabedian model and the global health security index as the theoretical frameworks to evaluate preparedness. The findings reveal that Haiti faces challenges in tackling the COVID-19 pandemic due to a lack of biosafety and biosecurity regulations, inadequate laboratory systems for COVID-19 testing, and shortages of human resources and personal protective equipment. Moreover, poverty remains widespread, and people lack access to clean water and sanitation services, resulting in a high risk of COVID-19 infection. Furthermore, a lack of communication, rumors, the circulation of fake news regarding COVID-19, and stigmatization cause distrust and reduce the number of people seeking healthcare services. Haiti faces challenges with respect to tackling the pandemic. The Haitian government can strengthen and improve the capacity of the healthcare system to fight against the COVID-19 pandemic and infectious diseases emerging in the future.

## 1. Introduction

Coronavirus disease 2019 (COVID-19) is an easily transmissible infectious disease that is transmitted through respiratory droplets among people with close contact, by aerosols, and by infected surfaces or objects [1]. To date, COVID-19 has affected more than 200 countries and territories worldwide. There have been approximately 540 million confirmed cases and 6 million deaths since 2019 [2]. The pandemic has resulted in the healthcare systems of developed countries being strained and facing shortages of resources, such as personal protective equipment (PPE) and ventilators [3,4]. To date, attention has been given to the efforts of developed countries to address COVID-19, although low-income countries are more vulnerable to the COVID-19 pandemic due to resource shortages [4] and fragile healthcare systems [5].

Haiti is the third-largest country in the Caribbean by area, with a population of 11,680,593, of whom 56.9% live in urban areas [6]. The Haitian population is very young—as the median age is 24, and the life expectancy is 65 years [6,7]. Haiti was the first black nation to gain independence in Latin America and has experienced intermittent attempts at democracy and recurrent periods of political instability. Haiti was one of the last countries to be affected by COVID-19. To date, there have been only 31,054 confirmed COVID-19 cases and 837 confirmed deaths in Haiti [8,9]. Additionally, it has performed 203,660 COVID-19 tests (4.66% positive results) [10]. Furthermore, Haiti was the last country in the Americas to initiate a COVID-19 vaccination program. To date, only 1.15% of the Haitian population has been fully vaccinated [11].

In addition, Haiti is the poorest country in the Americas and one of the most unequal in terms of access to health [12]. Given the fragility of the Haitian healthcare system, its vulnerability to the COVID-19 pandemic is alarming [13]. A previous study [14] reported that Haitian healthcare capacity was insufficient to meet the need, estimating that only 124 intensive care unit beds and 64 ventilators were available for the whole population before the COVID-19 outbreak. Furthermore, serious human resource shortages in the healthcare sector have exacerbated the response to the COVID-19 pandemic in many countries, including Haiti [15,16]. The Haitian healthcare system’s unpreparedness to cope with the COVID-19 pandemic is a great concern [13].

There is a paucity of research exploring the functional capacity and preparedness of the Haitian healthcare system against the COVID-19 pandemic. This study aims to assess the functional capacity and preparedness of the Haitian healthcare system regarding the COVID-19 pandemic by using the Donabedian model [17]. This model is regarded as the dominant paradigm for evaluating and improving healthcare quality [18]. It consists of three components: structure, process, and outcomes. The structure has a direct influence on the process, and the process also influences outcomes [19]. The Donabedian model helps analyze the resources used and their effectiveness during this pandemic in the current study.

Since COVID-19 is a new disease, an assessment of the relative appropriateness and effectiveness of health services can highlight the challenges and needed changes. The Global Health Security (GHS) index [20] was used to discuss the results of the current study and highlight the capacity, preparedness, and weaknesses of the Haitian healthcare system to prevent, detect, and respond to the ongoing COVID-19 pandemic.

## 2. Materials and Methods

### 2.1. Theoretical/Analytical Framework

The Donabedian model was employed as the theoretical framework to analyze the resources used and their effectiveness. Additionally, the GHS index was used to evaluate the capacity and preparedness of the Haitian healthcare system to cope with the ongoing pandemic and determine what is needed and what can be changed.

### 2.2. The Donabedian Model

According to the Donabedian model proposed by Avedis Donabedian, the quality of healthcare is based on three elements: structure, process, and outcomes [17]. Structure refers to all the factors of care delivery, including physical facilities (hospitals and centers), equipment (beds, lab tests, ventilators, and oxygen), and human resources (doctors, nurses, and support staff) [17,19]. Important aspects of human resources include the size of the workforce, degree of qualification, and organization, such as how staff are trained and paid (funding).

All activities of healthcare professionals are directly or indirectly related to prevention strategies, diagnosis, and patient treatment [17,19]. The model emphasizes technical and interpersonal aspects of how care is delivered (bed occupancy, admission procedures, and treatment) [19]. Outcomes can be assessed by changes in health status, behavior, or knowledge, including patient satisfaction and health-related outcomes, such as mortality rate, morbidity, complications, disability, and responsiveness [19].

### 2.3. The Global Health Security (GHS) Index

The Nuclear Threat Initiative and Johns Hopkins Center for Health Security, in collaboration with The Economist Intelligence Unit, developed the GHS index, aiming to set a high level of preparedness against epidemics that can lead to pandemics (https://www.ghsindex.org/report-model/ (accessed on 29 October 2020)) [20]. In this study, six categories of the GHS index, namely, prevention, detection and reporting, rapid response, health system, compliance with international norms, and risk environment, were used to evaluate the Haitian healthcare system’s preparedness for the COVID-19 pandemic.

### 2.4. Data Collection

This study was conducted from September 2020 to March 2021 and used a narrative review approach with an exploratory and evaluative purpose. The collected data were secondary data from administrative resources, such as financial tracking, databases, records (clinical records and COVID-19 curve), human resource information, policy data (Ministry of Public Health and Population of Haiti (MSPP) preparation system to fight the epidemic and World Health Organization (WHO)), facilities reporting systems (service readiness, quality, equity, risk protection, and health status), reports from national and international organizations (nongovernmental organizations (NGOs) and humanitarian organizations), journal articles, web pages, periodical articles, and online newspapers related to the current COVID-19 outbreak.

### 2.5. Data Analysis

Document analysis [21] was used to analyze the collected data based on the Donabedian conceptual framework. The analytical process consisted of researching, reading, and analyzing the documents. An interpretive analysis was needed to bring out the contents of the documents referring to the three dimensions (structure, process, and outcomes) and the GHS index indicators.

## 3. Results

### 3.1. Analysis of the Structures/Resources Used for COVID-19 in Haiti

#### 3.1.1. Physical Healthcare Facilities and Equipment

The MSPP developed a response plan against the COVID-19 outbreak in March 2020. According to this plan [22], in the interpandemic phase of preparation for COVID-19, two groups of isolation centers were to be established for clinical management: level 1 isolation centers (for noncomplex cases) and level 2 isolation centers (for complex cases). Fifty-nine level 1 isolation centers, with a total installed capacity of 577 beds, were to be installed in the 10 health services of the country. The majority of these isolation centers were former acute diarrhea treatment centers, which had adequate equipment. Fifteen level 2 isolation centers were to be installed in 10 departments across the whole country, with a total installed capacity of 143 beds, and were provided with the necessary facilities and an intensive care unit equipped with a respirator/ventilator, a heart monitor, equipment for gas measurement, a defibrillator, and equipment for measuring central venous pressure.

The epidemiological surveillance report published by the MSPP [23] mentioned that only 37 COVID-19 treatment centers were operational in Haiti, not 74 centers (59 level 1 centers and 15 level 2 centers), as the MSPP had announced in its response plan to COVID-19. Nevertheless, one nationwide survey showed that Haiti had an estimated 124 intensive care unit beds and 64 ventilators for the whole country [14], indicating fewer than six ventilators per million inhabitants. In September 2020, 37 ventilators were given to Haiti by the U.S. Agency for International Development [24].

#### 3.1.2. COVID-19 Surveillance Resources

A recent epidemiological surveillance update by the MSPP [23] provided data about institutional surveillance, detection points in hospital structures, and posts set up at entry points (airports and land borders). The data were transmitted through the epidemiology services of health departments before reaching the Department of Epidemiology of Laboratories and Research (DELR) for compilation, analysis, and publication. One report published in June 2020 suggested that Haiti had only two laboratories with the capacity to test for COVID-19 [25]. However, at present, four laboratories can perform COVID-19 PCR tests and antigen tests, three laboratories can perform COVID-19 PCR tests, and nine laboratories can perform COVID-19 antigen tests in the Center, West, and Artibonite departments across the county [26]. Additionally, 49 investigative teams and 310 contact tracing teams are active to prevent the spread of COVID-19 throughout the country [27]. Furthermore, a medical center has been established to follow the in-home isolation of suspected and confirmed COVID-19 cases [27].

#### 3.1.3. COVID-19 Prevention Resources

Since the start of COVID-19, more than 3.9 million people have received awareness messages through social media campaigns, distribution of leaflets and posters, dissemination of messages and nutritional information, sound trucks, radio, television, and town criers in the most remote areas [27]. Community awareness and prevention activities with the engagement of more than 10,000 community and religious leaders have been involved in the response to COVID-19 [27]. Additionally, some musical artists and groups have released music videos about COVID-19 and the appropriate practices to prevent the spread of the virus [27]. In total, 8700 hand-washing points were installed by projects funded by the United Nations International Children’s Emergency Fund (UNICEF) [27]. Other NGOs, such as Compassion, Zanmi Lasante, and the United Nations Office for the Coordination of Humanitarian Affairs (OCHA), in partnership with the MSPP, provided hygiene kits to schools and vulnerable populations [27,28,29]. Thirsty-six thousand N95-type masks and 1.1 million surgical masks were allocated to the General Directorate of Civil Protection for distribution [27].

#### 3.1.4. Financial Resources

Haiti was one of the first countries to receive emergency financing through the COVID-19 Fast Track Facility. Two weeks after the first two confirmed cases of COVID-19 in Haiti, the World Bank approved the COVID-19 Response Project for 20 million USD [30]. The Central Intervention Fund for Humanitarian Emergencies allocated USD 4 million to support work to fight COVID-19 in the health and drinking water, sanitation, and hygiene sectors (WASH) [31]. Additionally, a press release in April 2020 declared that the International Monetary Fund (IMF) had approved USD 111.6 million in emergency financing to help Haiti address challenges related to the COVID-19 pandemic [32]. The International Development Bank (IDB) announced that it would reassign USD 27 million with the aim of supporting Haiti’s healthcare system in reducing the impact of the pandemic [33]. The MSPP COVID-19 pandemic response plan required USD 37.2 million [22], and according to the humanitarian fund plan of the MSPP, increased funding amounting to USD 176 million was required for the detection and control of infections in health facilities, along with access to care and improved laboratory testing capacity to identify those in contact with confirmed cases [22]. For non-health-related actions, including responses to the indirect impacts of COVID-19, additional funds amounted to USD 39.3 million [22]. However, to date, an amount of 24.9 million has already been funded according to the revised humanitarian response plan of October 2020, which covers, on average, 17.2% of the total financial need for the COVID-19 health and nonhealth plan [34].

#### 3.1.5. Human Resources for COVID-19

The scarcity of healthcare workers was already an important issue for the Haitian healthcare system before the pandemic. There are only 0.234 physicians and 0.68 nurses per 1000 of the population [35]. By June 2020, 194 healthcare workers had been infected by COVID-19 [36]. As part of the response to the COVID-19 pandemic, given the shortage of the healthcare workforce, 5500 community health workers, including community health nurses and nursing assistants, were trained in and assigned to tasks such as contact tracing and surveillance, representing the largest category of health workers in the country [36]. Additionally, 1830 health care workers (medical staff and support staff) across the country were trained in how to use PPE appropriately. Furthermore, the lack of personnel to operate the new ventilators was addressed during the pandemic, and 642 medical staff were trained in providing oxygen therapy [37]. To meet the need for health professionals in the fight against COVID-19, the MSPP launched a registration campaign for doctors, nurses, auxiliaries, and other support professionals. Eighty new health professionals were recruited and distributed to different COVID-19 care sites [36].

### 3.2. Analysis of the Process and Results (Use of Resources and Outcomes)

#### 3.2.1. Haiti’s COVID-19 Situation

To date, all 10 departments located across Haiti have reported cases of COVID-19 [38]. The Haitian government acted quickly after the confirmation of the first two cases in March 2020; it halted all commercial passenger flights and instituted a 14-day quarantine for all entrants at home or in places prepared by the government for people with no family or houses in Haiti [39]. Two hotels located in the capital were intended for quarantine (Visa Lodge, Servotel), but the managers of these hotels said that they “never had a contract with the government on this subject and never received people in view of the quarantine required by the government” [40]. All quarantine cases in Haiti underwent in-home quarantining; however, according to the data collected, quarantine and social distancing were not respected in Haiti. For example, six people may live in one small home, making it difficult to maintain social distancing [41]. Additionally, most of the Haitian population relies on the informal economy to survive, and visiting public markets and using public transportation are daily routines [42]. Even though the government banned public gatherings and factory production, in reality, people were unable to comply with this policy. In Haiti, rumors and misinformation about COVID-19 circulated, and those who wore masks or respected quarantine measures often experienced pressure from others, particularly in rural areas [27].

Although the government required people to wear masks in public, masks were considered a luxury, since approximately 60% of Haiti’s population lives under the poverty line [43] and struggles with hunger every day. Food insecurity has been a growing concern in developing countries during the pandemic. It is estimated that more than 4 million people in Haiti have been facing acute food insecurity since COVID-19 began [44].

By 19 March 2021, Haiti had had 12,686 confirmed cases and 251 deaths, and more than 10,443 people had recovered [45]. Of the 11.8 million inhabitants, only approximately 53,000 people had been tested [46]. The relatively low number of confirmed cases might indicate an underestimation because of the low number of tested people. In addition, theories and ongoing investigations have indicated that the youth of the Haitian population, the climate (warm weather), and cross-immunizations might be causes for this low number of cases [47]. Most of the rural population lives more than 5 km from a health institution [48], and people are more willing to use traditional medicine to treat themselves than to be tested for COVID-19 [49]. More than half of the 8000 patients with confirmed infections refused to be hospitalized and returned home after the diagnosis, which resulted in the closure of several COVID-19 treatment centers and left some centers with only two or three patients [50].

#### 3.2.2. The Situation of Haiti’s Healthcare System during COVID-19

Disruptions in the healthcare system are currently being felt across the country [27]. There is great concern about some health indicators in Haiti. There was a 332% increase in the number of confirmed cases of malaria compared to the same period last year [51]. Additionally, there were decreases of 74% in hospital deliveries and 67% in antenatal consultations [51]. Furthermore, vaccination for children declined during the COVID-19 pandemic; for example, there was a 14% decline in inactivated polio vaccination and an 8% decline in the first dose of measles and rubella vaccination [27].

The number of patients decreased by 25% in some maternity wards [52]. The primary healthcare for people affected by chronic diseases, such as hypertension, diabetes, and cancer, also decreased [49]. Additionally, Haitian people were reluctant to go to the hospital due to distrust, misinformation, rumors, fears, and stigma regarding COVID-19 [53]. They preferred to stay home and self-treat with traditional medicine [53]. Some people worried that Haiti’s healthcare system was fragile and unable to respond to the pandemic [54]. In fact, healthcare professionals were ill-equipped to protect themselves against the COVID-19 virus while dealing with daily stress [42].

#### 3.2.3. Border Surveillance

Haiti’s president declared the state of health emergency after the confirmation of the first two cases on 19 March. Prevention measures were announced, including the closure of airports, ports to passenger traffic, and the 50 crossing points between Haiti and the Dominican Republic (46 unofficial and 4 official) [55]. Although Haiti closed the border with the Dominican Republic, some unofficial crossing points still experienced increased movement [55]. After the beginning of the pandemic, voluntary and involuntary returns (expulsion) of Haitians to Haiti increased. Two hundred forty-seven Haitians were deported from the USA from April to June [56], and 91,819 voluntary returns were observed at the border between 17 March and 13 August 2020 [57].

On 30 June 2020, Haiti reopened airports and borders. All air passengers in Haiti currently must comply with certain measures, such as proof of a negative PCR or antigen test, temperature screening, possession of a health declaration card, and self-quarantine for 14 days [58]. The MSPP and its partners are strengthening the case detection, referral, support, and follow-up of suspected cases among migrants and returnees from the Dominican Republic [27]. Additional facilities for medical staff, quarantine, and WASH have been set up at the country’s main entry points [27]. Sampling sites have been established at five entry points between Haiti and the Dominican Republic (Malpasse, Cornillon, Anse à Pitre, Ouanaminthe and Belladère) [23].

## 4. Discussion

To our knowledge, this study is the first to assess the Haitian healthcare system’s preparedness and functional capacity for the ongoing COVID-19 pandemic. The findings may be invaluable for developing or improving the preparedness of the Haitian healthcare system against potential pandemics of emerging infectious diseases (EIDs) in the future. We employed some indicators of the GHS index to discuss the results and address the weaknesses of the Haitian healthcare system against the pandemic.

### 4.1. Capacity to Prevent EIDs

#### Biosecurity and Biosafety

This study revealed that Haiti does not prioritize biosafety and biosecurity, since there are no regulations in this area, and all such efforts have been carried out only since the pandemic began. The 2019 GHS index showed that Haiti scored 0.0 for biosafety and biosecurity [59] because there was no preparation for preventing the appearance or reappearance of EIDs. It is also important to note that there were difficulties in transporting specimens to laboratories capable of testing for COVID-19, and the testing capacity was very limited, which affected the case-based analysis.

### 4.2. Capacity to Detect and Report EIDs

#### 4.2.1. Laboratory Systems

The findings showed that in terms of laboratory systems (scored 50.0 GHS) [59], before the onset of the COVID-19 pandemic, Haiti had only two laboratories with the capacity to perform the COVID-19 PCR test, both of which were located in the capital (Port-au-Prince). This highlights the limited capacity of the laboratory systems in terms of number, infrastructure and equipment, human resources, and centralization. However, the study also found that efforts have been made to reduce centralization and increase the number of laboratories, infrastructure and equipment, and human resources during the pandemic. According to the WHO, one of the crucial elements for the response to COVID-19 or any other infectious disease are good laboratory practices. Laboratories with all the necessary equipment for quality control and accurate results, trained personnel, and biosafety and biosecurity, are also essential [60].

#### 4.2.2. Real-Time Surveillance and Reporting and an Epidemiological Workforce

This study found that real-time surveillance and reporting structures are present throughout Haiti; however, the surveillance systems were not ready at the beginning of the pandemic, and some are still not ready. Additionally, the results showed evidence of nonofficial border crossings, and people continued to enter the country without being reported. In addition, regarding self-reported cases, the decreasing number of people going to health facilities due to distrust and misinformation diminished the quality of the real-time surveillance. Strengthening real-time surveillance, including daily data collection and reporting, in Haiti, to facilitate communication and collaboration across sectors and at the national and international levels, has been crucial during the COVID-19 pandemic [61]. Overall, Haiti’s detection capacity received a score of 48.3 [59], and its laboratory systems and epidemiological workforce still need to be improved to prepare for potential EID pandemics in the future.

### 4.3. Capacity to Respond Rapidly to an EID Outbreak

#### 4.3.1. Emergency Preparedness and Response Planning

According to the GHS index, Haiti does not have a public health emergency response plan in place, and it scored 0.0 on this indicator [59]. This study revealed that the response plan to the pandemic was developed after the WHO declared COVID-19 a pandemic; therefore, there was no time to properly design and test it. The plan changed at each stage of the outbreak’s development. According to the WHO, having an emergency preparedness plan reinforces the capability of a country’s government, institutions, and communities to manage all types of disasters (natural or health-related). This plan must be in place, tested, and modified depending on the disaster [62]. Emergency preparedness is a continuous process that relies on the collaboration of communities at the local, subnational, and national levels to effectively manage an emerging or current public health emergency [63].

#### 4.3.2. Risk Communication and Access to Communication Infrastructures

The results also showed that the public health sector used all kinds of communication tools (the MSPP and DELR websites, social media, statements by public and religious figures, and radio and TV broadcasts) to inform the population about the pandemic outbreak; however, more than half of the population was either uninformed or misinformed about COVID-19.

In Haiti, distrust of the government obstructed efforts to prevent the spread of the virus. Risk communication with the public is an essential point in the management of public health emergencies [64], as it can mitigate risks, support the actions that need to be taken, and reduce the negative health impacts on the population. Previous studies have indicated the importance of effective risk communication to decreasing misconceptions and thereby influencing the population’s perceptions or promoting changes in personal protective behavior during a pandemic [65,66].

#### 4.3.3. Trade, Travel, and Border Control

The findings underlined the well-defined border controls and travel restrictions at the beginning of the outbreak in the country. Nonetheless, deportees from the USA and illegal entries at (unofficial) border crossings with the Dominican Republic were reported. Additionally, the early reopening of the borders for tourism and trade can be considered a failure of the surveillance system because afterward, new imported cases were reported.

Haiti’s scores for response capacity were very low, including in public health emergency preparedness, risk communication, and border control. According to the GHS index, response capacity is one of the most important indicators in the fight against a potential pandemic.

### 4.4. Capacity of the Health System

#### 4.4.1. Health Capacity in Clinics and Community Care Centers

The findings showed that the percentage of health workers per inhabitant in Haiti is very low; moreover, health workers have not been trained in the management and treatment of COVID-19. Haiti is one of the countries with a very low percentage of human resources in the health sector.

This study also highlighted the lack of protective equipment for these workers, which led to excessive fear among them. Many doctors and nurses expressed fear about returning to work if they could not protect themselves. This fear could also be explained by the lack of communication between the responsible entities (the MSPP) and health workers; without training, protective equipment, and adequate communication, liabilities in the management of the pandemic were very likely. The results also indicate that training and recruitment were carried out, which should have reduced the shortage of human resources for the management of COVID-19.

The results for the number of COVID-19 isolation and management centers are uncertain. According to the findings, less than half of the centers planned for the management of COVID-19 cases were operational; and the majority did not have the necessary infrastructure and equipment, such as ventilators, oxygen for severe cases, WASH systems for hygiene, and protective equipment for workers, patients, and visitors. There was also uncertainty regarding quarantine centers, which, according to the findings, did not exist even for tourists, with only home quarantine being carried out.

Despite all these observed gaps, the existing treatment centers were almost empty because of the population’s distrust and lack of education about COVID-19.

#### 4.4.2. Healthcare Access

Haiti established a law making universal health coverage mandatory in 2005 [67], but according to the findings, only 60% of the population had access to health services, and most services were located in metropolitan areas [68]. The findings revealed that Haitian people who tested positive for COVID-19 preferred to return home rather than being hospitalized, and others, even though they had symptoms, preferred not to be tested. These choices stemmed from the distrust and misinformation that came from false rumors. People in Haiti trust shamans and pastors more than the government. Furthermore, most Haitian people use so-called natural or traditional medicine. These measures prevent people from seeking health services and decrease vaccination coverage, the use of maternal and child health services, and the use of chronic disease health services, which may lead to a severe health disaster in the future.

### 4.5. Compliance with International Norms

#### Financing

The results showed that the financing of the Haitian healthcare system depends largely on external partners, such as NGOs and the World Bank. Without these partners, Haiti would not even have a healthcare system. After COVID-19 cases were observed in Haiti, millions of dollars were provided to Haiti for the management of COVID-19. Some of these funds were non-COVID-19 funds intended to mitigate the negative impacts of international and national measures on the implementation of the response already planned (prepandemic and postpandemic) for people and services previously targeted in the initial humanitarian response plan.

Over 150 NGOs working in all fields in Haiti disbursed millions of dollars to improve the WASH system, food insecurity, and maternal and child health; however, Haiti is still considered the unhealthiest and poorest country and has the highest rates of maternal and child mortality. Good management of the healthcare system does not rely on NGO funding. This shows that while Haiti, a poor country, needs help, it also needs more financial independence and management regarding health matters.

### 4.6. Risk Environment

#### 4.6.1. Socioeconomic Resilience

The findings also highlighted people’s noncompliance with the health standards proposed by the government due to their distrust of the government. Another reason may be the economic situation in Haiti: most of the population lives on a daily income (less than USD 2 per day); and market closures, social distancing, and stay-at-home measures resulted in no income and no food for their families. Food insecurity has been a great concern in Haiti during the COVID-19 pandemic and needs urgent action.

#### 4.6.2. Public Health Vulnerabilities

The findings also showed that most Haitian people are still suffering from poverty, without clean water and sanitation services, which results in a high risk of becoming infected with COVID-19. In addition to lack of water and sanitation, access to high-quality healthcare is limited among the Haitian population. People fear that the fragile healthcare system cannot deal with COVID-19. Some Haitian people prefer self-treatment and use traditional medicine rather than going to hospitals.

## 5. Limitations and Future Research Direction

This study has the following potential limitations. First, it used mainly secondary data, so the documents could be biased. Primary data collection methods could potentially yield different results. Future research can be carried out through face-to-face interviews or on-site data collection, which might help to better understand the issues. Second, the information on the funding and preparedness of the Haitian healthcare system during the pandemic is not complete, as most of it is undocumented and unavailable for use in academic research. Public documents are not easily accessible, and much information is missing in this study. In the future, researchers could make an effort to cooperate with the Haitian government to ensure the availability of official documents. Third, this study was carried out between the sixth and seventh months of the pandemic, while the numbers of confirmed cases and deaths, funding, etc., were continuously increasing and preparedness plans were changing. Further studies could be carried out after the pandemic to assess the preparedness, effectiveness, and outcomes of the pandemic in Haiti and to explain the discrepancies between the expectations of scientists and reality. Other studies could focus on inequality in access to healthcare, universal health coverage, and the lack of trust and communication between responsible health entities and the population.

## 6. Conclusions

Haiti is one of the poorest countries in the Caribbean; it is the most inequitable in terms of healthcare and is vulnerable to natural disasters. This study showed that Haiti faces challenges with respect to tackling the pandemic. The Haitian government can strengthen and improve the capacity of the healthcare system to fight against the COVID-19 pandemic and infectious diseases that will emerge in the future. Additionally, considering the weakness of public health emergency preparedness and response capabilities in Haiti, effective international collaboration is critical to help Haiti fight against the pandemic.

## Data Availability

All relevant data are within the paper.
